# The British Journals: Medicine

**Published:** 1839-04

**Authors:** 


### MEDICINE.

### *On Tubercles in the Brain of Children.* By P. Hennis Greene, M.D.

[This forms another of those admirable "Contributions to the Pathology of Children," for which we have been, during the last two years, indebted to Dr. Greene, and which we hope to see made the foundation of a separate treatise. The present paper contains four cases, with the appearances on dissection. We can only find room for some of the *Remarks* appended to them.]

It is a curious point in the history of medical literature that, although tubercles of the brain constitute one of the most frequent and important affections of the nervous system in children, they have been neglected by all systematic writers on diseases of children, without exception. I am acquainted with almost every work of any reputation on children's diseases in all European languages, and cannot call to mind a single one in which even a few lines, much less a chapter, are devoted to the consideration of cerebral tubercles. This cannot arise from the comparative rarity of tubercles of the brain in children, for a long and laborious investigation of this interesting subject enables me to affirm that, in point of frequency, they must be ranged next to hydrocephalus; and that for every three cases of the latter disease, there exists one of the former. On the other hand, tubercles of the brain are extremely rare in the adult subject. M. Louis examined 117 bodies of adults who had died of pulmonary consumption without meeting with more than one example; and all who have been in the habit of making numerous post-mortem examinations will remember how seldom this lesion of the nervous centres in adults presents itself to the pathologist. The theses of some of the French *internes*, however, contain a mass of valuable information on tubercles of the brain, amongst the best of which may be mentioned those of MM. Mitivié, Giraud, Tonellé, Leveillé, and Dufour.

Tubercles of the brain often exist without producing any disturbance of the cerebral functions, and are only discovered on examination of the body after death. Of this I have seen frequent examples. The foreign body may even attain a very considerable size without giving rise to irritation or inflammation of the surrounding nervous tissue; and unless situate in the neighbourhood of some of the principal nerves, or important divisions of the brain, the pressure which it must necessarily exercise seems, as it were, to pass unheeded by the centre of the nervous system. In other cases a small tubercle will excite more or less irritation and congestion of the surrounding substance, and develop a train of symptoms which, although frequently obscure, still present a certain character or order from which the nature of the lesion may be discovered.

In the present remarks I do not propose to enter, at any length, into the history of cerebral tubercles in children, but merely to direct attention to a few of the leading symptoms which presented themselves in the cases above described.

Headach, more or less intense, either perpetual or remittent, sometimes occupying the frontal region, at other times corresponding exactly with the seat of the tubercle, is a very frequent symptom. It existed in all the cases which I have related. In that of Masson, where the tubercle occupied the posterior part of the cerebellum, the headach was principally fixed in the back of the head and neck.

Chronic vomiting, occurring at uncertain intervals, and not apparently connected with disorder of the alimentary canal, is another symptom which constantly exists, and is of great value in the diagnosis of cerebral tubercle, when conjoined to headach and constipation of the bowels. The latter symptom is one to which too much attention cannot be paid. It may depend, of course, upon a variety of causes; but long-continued and obstinate constipation in children is generally, as far as I have observed, connected with some organic lesion of the nervous centres, and more especially with chronic meningitis or tubercle.

Some disorder of the motor power, manifested by irregularity of the gait, an incapability of harmonizing the movements, partial paralysis, or a contracted state of one of the limbs, often exists as a symptom of cerebral tubercle. It preceded the acute symptoms for some time in the case of Jane Masson; and I have frequently noticed a similar phenomenon both in cases of tubercle and of chronic meningitis, the forerunner of hydrocephalus. The intellectual functions are seldom disturbed at an early stage of cerebral tubercle; but as the disease advances, more or less change either in the temper of the child, the memory, or some other mental faculty, usually manifests itself; irregular accesses of fever (which is often mistaken for infantile remittent fever) occur, with delirium at night; and in some instances the patient is gradually reduced to a state of complete idiocy.

Tubercles of the brain in children commonly destroy life, either by inducing acute hydrocephalus, or by exciting inflammatory softening of the surrounding nervous tissue. Indeed, the relation between acute hydrocephalus and tubercle is much more close than has been generally admitted. On some other occasion I shall endeavour to determine the precise relation, by the statistical records which I possess; but my impression is that tubercles of the nervous centres exist in one fourth to one sixth of the cases of hydrocephalus which occur in children above twelve months of age.

The influence of age on the production of cerebral tubercle is very remarkable. On looking over a list of 42 cases which I have observed within a period of five years, I find that there occurred—

| | | | |
|---|---|---|---|
| At the age of 1 year ...... | 1 | | 38 |
| 2 ............ | 11 | At the age of 9 years...... | 0 |
| 3 ............ | 5 | 10 ............ | 2 |
| 4 ............ | 5 | 11 ............ | 0 |
| 5 ............ | 8 | 12 ............ | 1 |
| 6 ............ | 2 | 13 ............ | 0 |
| 7 ............ | 5 | 14 ............ | 0 |
| 8 ............ | 1 | 15 ............ | 1 |
| | 38 | Total... | 42 |

Of 38 other cases which have been observed by different *internes* at the Children's Hospital, Paris, there were

| | | | |
|---|---|---|---|
| At the age of 1 year ...... | 0 | | 30 |
| 2 ............ | 0 | At the age of 9 years...... | 3 |
| 3 ............ | 8 | 10 ............ | 4 |
| 4 ............ | 5 | 11 ............ | 0 |
| 5 ............ | 5 | 12 ............ | 0 |
| 6 ............ | 6 | 13 ............ | 1 |
| 7 ............ | 3 | 14 ............ | 0 |
| 8 ............ | 3 | 15 ............ | 0 |
| | 30 | Total... | 38 |

Hence, in a series of eighty cases, we find the maximum number to occur between the ages of two and four years inclusive. I have already shown, in another paper (*Lancet,* vol. ii. 1835-6,) that acute hydrocephalus occurs most frequently between the ages of five and seven years.

The diminution in the number of cases after the second dentition, which my own table exhibits, is very remarkable, and worthy of notice. Scarcely a case occurred after the age of seven years.

In the preceding remarks I have merely indicated a few of the leading points connected with a lesion which has been, as I have already observed, entirely overlooked by writers on the diseases of children. *Lancet. Feb.* 16, 1839.

---

### *Attempt to cure Elephantiasis by the poison of the Rattlesnake.*
### By R. W. Clarke, Esq., Assist. Surg. R. N.

[This case is very remarkable, and merits record on many accounts. The minute accuracy of the details is much to be commended.]

Masianno Jose Machado was bitten by a rattlesnake, for the express purpose of being cured of elephantiasis and lepra. The individual who made this experiment* was a white man, about fifty years of age, of ordinary stature, stout, and rather athletic in form; temperament, sanguineo-bilious. The kind of elephantiasis with which he was affected was that denominated by Alibert, E. leontina; it was in its second stage, and, according to the patient, no energetic measures had been employed in its treatment. Nearly all his body, as well as extremities, was insensible exteriorly; the cutaneous tissue was thickened, hard, its surface rugous, covered with tuberculous elevations, but none of them were ulcerated. Some pustules under the arms had a porriginous appearance. The characters of the disease were more apparent, and better developed on the face, the features of which were swollen, giving a disagreeable aspect, without, however, rendering it altogether hideous. On the extremities the skin and nails had begun to change in appearance, and the fingers and toes were altered in form. Whilst life and sensibility appeared almost extinct on the surface of his body, his interior yet retained the remains of his former energy, and he possessed a force of mind by no means common, and seldom found in one of his sad condition. Six years of

* The experiment was suggested by a case which had been reported from the interior, where an accidental bite of the rattlesnake had terminated favorably.

dreadful and incurable disease, and four of seclusion in the hospital for lepers, had made him look forward to death as the only termination to his sufferings. No danger counterbalanced, in his idea, the desire he felt to be freed from his disease; he willingly risked the remainder of a life, under its continuance, for the slightest probability of recovery; and no Stoic ever expired more undaunted and indifferent than he did when aware of the fatal effects of his experiment. No opinion had the least weight against the determination he had taken; nothing intimidated, nothing deterred him. Having obtained leave to quit the Lazarus Hospital, he resolutely repaired to the house of Sen. Santos, physician to the hospital, to offer himself to the fangs of the venomous reptile, whose bite sometimes destroys life in a few instants, causing, immediately, tremors, convulsions, and the blood to issue from the different outlets of the body, and even from the pores of the skin. Having signed a declaration that the act was voluntary, and that he himself was alone responsible for its results, he boldly introduced his hand into the cage of the deadly reptile; it at first appeared to avoid him; he advanced his hand towards the snake: it looked inoffensively at him, and began to lick his hand. Two minutes passed in this repugnance on the part of the reptile to bite him. He now provoked the serpent, and seized it in his hand forcibly, and it bit him between the articulations of the ring and little fingers with the metacarpus. The bite was inflicted at 50 minutes after 11 in the morning of the 4th September. He felt no pain when bitten, nor effects from the poison introduced into the wound; he only knew that he was bitten when it was announced by the bystanders.

His hand was immediately withdrawn from the cage; it swelled slightly, and a few drops of blood escaped from the wound, but he felt no pain. The man continued perfectly tranquil; respiration natural, and his pulse regular. Five minutes after the bite, a slight sensation of cold in the hand.

12, noon. Slight pains in the palm of the hand, which increased after some minutes.

17 minutes past 12, noon. The pain extended to the wrist.

20m. The hand swelled considerably.

30m. The pulse became fuller. The patient all this time conversed in a lively manner, and even laughed.

50m. A sensation of fulness in the course of the jugulars; some alteration in vision; formication in the face.

55m. The sensation of fulness extended to the sides and back part of the neck; the hand continued to increase in volume, and the pain extended two thirds up the forearm.

59m. Numbness over the whole body.

1h. 20m. P. M. Tremor of the whole frame; sensible to the touch.

36m. Cerebral disturbance; pulse more frequent: some difficulty in the movements of the lips; somnolency; sensation of constriction in the throat; pain more intense, and extending over the whole arm; increased intumescence of hand.

38m. Felt cold, and covered himself.

48m. Pain in tongue and fauces, extending down to the belly; increased pain and swelling in hand; coldness of feet.

2h. 5m. Difficulty of speech.

25m. Difficult deglutition; anguish; copious perspiration on the chest.

50m. Arms powerless; some drops of blood from the nose; increased anguish and inquietude; pulse 96.

3h. 4m. General swelling; involuntary groans; sensation of sinking.

8m. Pulse 100.

15m. Great pain in the arms; restlessness.

30m. Pulse 98; flushed face; continued bleeding from the nose.

35m. Drank a little wine and water without difficulty; his shirt was changed, wet with perspiration; intense redness of the whole body; some drops of blood escaped from a pustule under the arm.

4h. Pulse 100; redness of surface more intense, but of a darker hue, especially in the bitten limb; violent pains in superior extremities, preventing any rest, notwithstanding the exhaustion of which he complained; constriction of throat, and breathing embarrassed; inferior extremities and belly as yet not exhibiting any particular phenomena.

50m. Pulse 104; great heat over the whole surface of the body; salivation.

5h. 30m. Pulse in same state; torpor. It is remarkable that the urine has all along flowed in great abundance; saliva viscid, of a dark colour, and expectorated with difficulty; great muscular prostration; frequent groans, caused by pains over whole body; respiration tranquil; pulse full; skin soft; increased tumefaction of bitten hand. In this state he continued till

7 P. M. Some disturbed sleep, with groans; he woke, and said he was free from pain in the arms, but had great pain in chest, and a feeling as of a knot in the throat; urine copious; deglutition very difficult; saliva viscous and white; sanguinolent fluid running from the nostrils; offered a drink of water, with rum and sugar, which he could not swallow.

8h. Sweating ceased; groaning not so constant.

30m. Passed urine.

9h. 10m. Passed urine; ceased to groan.

15m. Profound sleep.

10h. Administered the infusion of guaco; dose, three tablespoonfuls, with one of eau-de-luce, which patient refused, and took the simple infusion; sanguinolent secretion from the nose stopped; pulse regular; diminution of the tubercular elevations of both arms and face, presenting an appearance of erysipelatous redness.

20m. Patient passed about two ounces of tolerably perfect urine; remains more tranquil, and sleeps at intervals without groaning.

40m. Pains in chest diminished; pains in legs and feet, in which, until this time, there had been a sensation of death-like cold; pulse regular, 108; thirst; patient drinks water without difficulty.

11h. Takes four tablespoonfuls of infusion of guaco.

45m. Urine high coloured; drinks water easily by spoonfuls; pulse 119; the wounded hand and arm inflamed, and very painful; restlessness.

Midnight. Slept soundly, interrupted by eructations; pulse 112; passed urine.

30 minutes past 12. Patient very restless; his cries distressing; calls for religious consolation; refuses medicine.

40m. Again passed urine; pulse 116; sensation of great heat in the legs; desires the coverlet to be removed.

1h. Patient takes his medicine again; asks to be uncovered; passes urine; becomes more quiet.

15m. Passes urine; pulse 100.

40m. Takes a dose of infusion of guaco.

2h. Drinks three spoonfuls of water; sits up in his bed; every time he drinks, pain and restlessness increase.

10m. Passes urine.

30m. Takes his medicine; becomes more tranquil.

3h. Passes urine; the lower lip, which had been much swollen and inflamed, returns to its natural state; salivation ceases.

55m. Passes urine, the quantity always from ℥ij. to ℥iij.; is more tranquil; takes his medicine; pulse 110; involuntary movements of right thumb and left leg.

4h. Passed urine.

45m. Takes a spoonful of medicine; pulse 100; patient tranquil, and sits up.

5h. Passes urine.

30m. Passes urine; patient declares himself in great agony.

6h. Pulse 100; respiration free; frequent groans.

10m. Passed urine.

9h. 15m. Great prostration; convulsive movements of the lower jaw, as also of the lower extremities; urine bloody.

10h. Pulse accelerated and intermitting; increase of convulsions; diminution of swelling of limbs, and redness of skin; deglutition extremely difficult; respiration anxious. Applied blisters to the thighs; gave a spoonful of infusion of guaco.

50m. Convulsions diminished; administered enema of brandy.

55m. Cessation of convulsions.

11h. Remains in same state. Gave an ounce of oil of Laga, which he swallowed with difficulty.

30m. The patient expired.

In a few hours the corpse became livid and more swollen; at ten the following morning, eleven hours after death, the body was enormously increased in volume, and covered with red and livid spots, exhaling a fætor so insupportable as to preclude the possibility of an autopsy, as we desired. *Lancet. Dec.* 15, 1838.

---

*Revaccination at the Foundling Hospital.* By W. B. HUTCHINSON, Esq.

I HAVE recently submitted 216 children at the Foundling Hospital to the test of revaccination; and, as this subject is just now creating so much interest amongst the members of the profession, it may be useful to lay before your readers the result of my observations upon these cases. Of these 216 revaccinated, 11 went through the different stages of regular cowpox, as if they had never had the disease, proving that in them the primary vaccine had lost its prophylactic power.

In 122 a spurious form of cowpox was developed, producing, in some, considerable local inflammation and constitutional disturbance. The irregular nature and progress of the spurious affection in all these cases proved that the constitution was still under the protective influence of the first vaccination.

In 83 no effect (more than slight irritation from the puncture) was produced, though many of these were revaccinated twice; thus affording negative evidence of the undiminished preservative power of the primary vaccine.

I was led, in the first instance, to recommend a general revaccination of the children, from observing that the arms of many of those who had recently arrived from the country (of the age of five years) presented very imperfect cicatrices; but I think that the following short observations will go far to prove that the condition of the cicatrix does not furnish any criterion whereby to estimate the success or efficacy of the early vaccination; and I feel justified in concluding that the intensity of the local inflammation produced by the vaccine virus bears no relation to its subsequent protective virtue.

Of the 11 cases revaccinated, in which all the steps of natural cowpox were regularly developed, 8 presented perfectly-formed cicatrices (some having four, none less than two); 2 presented no trace of a cicatrix; and 1 had an imperfect scar. The youngest of the 11 was aged five years, the oldest aged thirteen years. I selected three arms, in neither of which could any trace of a cicatrix be discovered (though the children were all carefully vaccinated when infants), and I revaccinated them twice with the greatest care from a fine arm, without any result more than a slight degree of inflammation in one out of the three cases. Being desirous of employing only recent lymph from the infant's arm (for the greater security of the children revaccinated), I had no extensive opportunity of testing the lymph of those cases of successful revaccination; and the few experiments which I did make were inconclusive. *Lancet. Jan.* 19, 1839.

---

*Observations on the Exhibition of Mercury in minute Doses.* By ROBERT LAW, M.D., A.M., Physician in Ordinary to Sir Patrick Dunn's Hospital.

[THIS is a valuable communication, well deserving the attention of practitioners. The experience of every one of any standing in practice must have convinced him both of the carelessness with which mercury is generally exhibited, and of the great uncertainty of its effects under such circumstances. We have been long convinced of the impropriety of the large doses usually given of this medicine (as of many others); but we have nowhere seen the fact placed in so clear a light as by Dr. Law.]

There is not in the Materia Medica an agent whose just pretensions are more compromised by a slovenliness and want of care in its exhibition than mercury. And although, in some cases, the peculiar circumstances under which the medicine is exhibited prevent the conditions of restricted diet and confinement being complied with; and, therefore, the physician prescribes it under disadvantages, of which, although he is aware, yet he cannot control them; still we believe, from the little importance attached to these conditions when they might be enforced, that most physicians have yet to learn to what extent they modify the action and effects of the medicine. Upon inattention to these circumstances we should charge, in many instances, the complete failure of mercury to affect the system; and in all, that when the system is brought under its influence, so much more of it is required to produce this effect. An anxiety to ascertain to what extent a due attention to these modifying conditions would influence the results of the medicine, led us to bestow a good deal of pains upon the subject. An hospital, of course, afforded the best opportunity for such enquiry, as there alone could strict attention to directions be ensured. To a single result of such enquiry will we advert at present, viz. *the very small quantity of mercury required to affect the system, when exhibited in minute doses at short intervals.* This quantity was much smaller than we could have had any idea of. The first cases in which we made trial of this mode of giving mercury were chronic cases, such as we felt would, without injury or detriment, await the result of our experiment. We made no particular selection of cases, but such as were labouring under affections which we ordinarily treated with mercury. We directed one grain of calomel to be mixed up with a sufficient quantity of extract of gentian to make a mass to be divided into twelve pills, one of which was to be taken every hour. We found, in some cases, salivation produced by twenty-four pills, or two grains of calomel; and seldom were forty-eight pills, or four grains, required to produce this effect. We would say, that thirty-six pills, or three grains, was the average quantity required to effect salivation. We exhibited blue pill in the same way, and found the mouth to become sore from six grains.

We have not yet tried it in primary syphilis. But this we have observed, that we have succeeded in bringing the system under the influence of mercury in a very short time by frictions of ten grains of the ointment. We have found one drachm divided into six parts, and one part rubbed in every night, sufficient to produce salivation.

[Several cases are given, illustrating the good effects of the remedy, employed in this manner; and, among the rest, two in which the constitutional effect was *postponed* by the patients taking a *larger* dose than was prescribed; in these two cases the medicine acted on the bowels. The following is Dr. Law's explanation of the facts.]

We conceive the efficacyof this mode of exhibiting mercury to depend not only upon its *remora* in the system being ensured by the smallness of the dose, but also, upon a succession of impressions being kept up by the exhibition of these small doses at intervals, not so distant as that the effects of the impression be passed away before they be succeeded by another; nor yet so short that the small doses, crowded upon each other, produce but one impression, and that one such as would result from a single dose, equal to the sum of the small doses.

[Dr. Law concludes as follows:]

It now remains to be established, if this effect from a small be equal to that produced by a larger quantity. Of this we feel quite confident, that experience will prove that all the advantage to be derived from the medicine is within the compass of a much smaller quantity than has hitherto been supposed, provided that small quantity be exhibited with due attention to circumstances calculated to promote its effect; and we would further expect that this more guarded exhibition of it would save us from the frightful mischief that we sometimes see following it when largely administered; and which sometimes suggest to us the question, if mankind would not have been benefited by an agent, capable of such mischief, never having been introduced into the Materia Medica.

*Dublin Journal of Medical Science. Jan.* 1839.

*On the Nervous Tremor of Children.* By P. HENNIS GREENE, M.D.

I do not remember to have seen this affection of the nervous system described in any English work on diseases of children, while, by several foreign writers it has been confounded with chorea. There is, however, a striking difference between the two diseases. In chorea the movements are involuntary and distinguished by a great degree of irregularity. The patient finds it impossible to execute certain motions in a regular manner. In cases of "nervous tremor" the movements are also involuntary, but are far from exhibiting the characteristic irregularity of chorea. They are rather marked by oscillations occurring from above downwards, or from side to side.

[Three cases are given which appear to have been observed in a French hospital, the practice being inert. We extract some of the *remarks* on them.]

In the first case, which terminated fatally, the whole of the central nervous system was examined with the greatest care, but no lesion could be discovered. The disease was probably occasioned by mental emotion, but the presence of contracture of the limbs, with shooting pains, and pain along the spine, was calculated to mislead the judgment, especially as the child suffered under pulmonary consumption.

In the second case, the nervous tremor was evidently occasioned by the derangement of the system previous to the establishment of the catamenial discharge; while in the third case, the exciting causes of the malady were complex, the most manifest being the influence of lead. The treatment of this latter case was excessively timid and defective; had the boy been freely purged on his admission into the hospital, it is evident that all the symptoms of the complaint would have been speedily dissipated. *Lancet. Dec.* 22, 1838.

